# Prg4-Expressing Chondroprogenitor Cells in the Superficial Zone of Articular Cartilage

**DOI:** 10.3390/ijms25115605

**Published:** 2024-05-21

**Authors:** Nadezda Ignatyeva, Nikita Gavrilov, Peter S. Timashev, Ekaterina V. Medvedeva

**Affiliations:** Institute for Regenerative Medicine, Sechenov First Moscow State Medical University (Sechenov University), 8-2 Trubetskaya St., Moscow 119048, Russia; gavrilovns2001@mail.ru (N.G.); timashev_p_s@staff.sechenov.ru (P.S.T.); medvedevaekaterina@yandex.ru (E.V.M.)

**Keywords:** articular cartilage, chondroprogenitors, lubricin, Prg4, superficial zone, regenerative medicine

## Abstract

Joint-resident chondrogenic precursor cells have become a significant therapeutic option due to the lack of regenerative capacity in articular cartilage. Progenitor cells are located in the superficial zone of the articular cartilage, producing lubricin/Prg4 to decrease friction of cartilage surfaces during joint movement. Prg4-positive progenitors are crucial in maintaining the joint’s structure and functionality. The disappearance of progenitor cells leads to changes in articular hyaline cartilage over time, subchondral bone abnormalities, and the formation of ectopic ossification. Genetic labeling cell technology has been the main tool used to characterize Prg4-expressing progenitor cells of articular cartilage in vivo through drug injection at different time points. This technology allows for the determination of the origin of progenitor cells and the tracking of their progeny during joint development and cartilage damage. We endeavored to highlight the currently known information about the Prg4-producing cell population in the joint to underline the significance of the role of these cells in the development of articular cartilage and its homeostasis. This review focuses on superficial progenitors in the joint, how they contribute to postnatal articular cartilage formation, their capacity for regeneration, and the consequences of Prg4 deficiency in these cells. We have accumulated information about the Prg4+ cell population of articular cartilage obtained through various elegantly designed experiments using transgenic technologies to identify potential opportunities for further research.

## 1. Introduction

Articular hyaline cartilage plays a vital role in the function of diarthrodial (synovial) joints. Chondrocytes, the sole cells present in hyaline cartilage, exhibit heterogeneity throughout the tissue structure. Articular cartilage (AC) comprises distinct zones [1,2,3,4]: a narrow superficial zone (SFZ) that provides boundary lubrication to protect the surfaces of the joint during its motion; transitional (middle) and deep zones, which resist against mechanical loads on the joint; and a calcified zone that connects AC to the subchondral bone [5,6,7]. The structure of AC is unique in that it has an avascular and aneural nature, low cell density, and restricted capacity for self-repair [8,9,10].

Until 2000, little was known about the growth mechanisms of AC. In 2001, to study AC’s postnatal development in 3-month-old marsupials, Hayes et al. [11] administered the thymidine analogue bromodeoxyuridine (BrdU) into the knee joint. This reagent is incorporated into DNA during cell division, blocking further cell cycling. The experiment revealed that chondrocytes occupying the transitional zone were depleted one month post-injection, leading to thinning of AC [11]. Furthermore, based on BrdU injection research [11], it was indicated that the flattened surface zone cells had a longer cell cycle time compared to the underlying transitional zone cells. Based on these results [11], Hayes et al. suggested that the flattened surface zone cells of AC might be a slow-cycling chondroprogenitor population.

This hypothesis developed through the identification of progenitor-like characteristics of a subpopulation of immature bovine SFZ chondrocytes in vitro [12,13]. It was reported that the cells from the SFZ of AC exhibited a high affinity for fibronectin and possessed high colony-forming efficiency. Transitional zone chondrocytes exhibited more affinity with fibronectin than SFZ cells (15% middle vs. 10% surface), but they lacked the ability to form colonies [12].

SFZ cells express the gene neurogenic locus notch homolog protein 1 (Notch1), which is the regulator of cell fate decisions [12,13], and, according to flow cytometry, 86% of the SFZ cells isolated by pronase/collagenase digestion were found to be positive for Notch1, compared with 10% and 34% of the middle and deep zones, respectively [12]. The treatment of AC chondrocytes with DAPT, the Notch signaling inhibitor, did not affect the adhesion of SFZ and deep zone cells to fibronectin but abolished the colony-forming efficiency of SFZ cells. Likewise, bovine cartilage explants cultured with DAPT exhibited a hypocellular zone below the articular surface, and BrdU immunolabeling revealed the absence of proliferation [12]. The superficial lineage of bovine AC was shown to have the potential to differentiate into AC as well as bone, tendon, and perimysium in the embryonic chick system [12,13]. The population of SFZ cells exhibited plasticity in their differentiation and were responsible for the appositional growth of AC tissue. However, there was no definitive marker yet.

**Figure 1:**
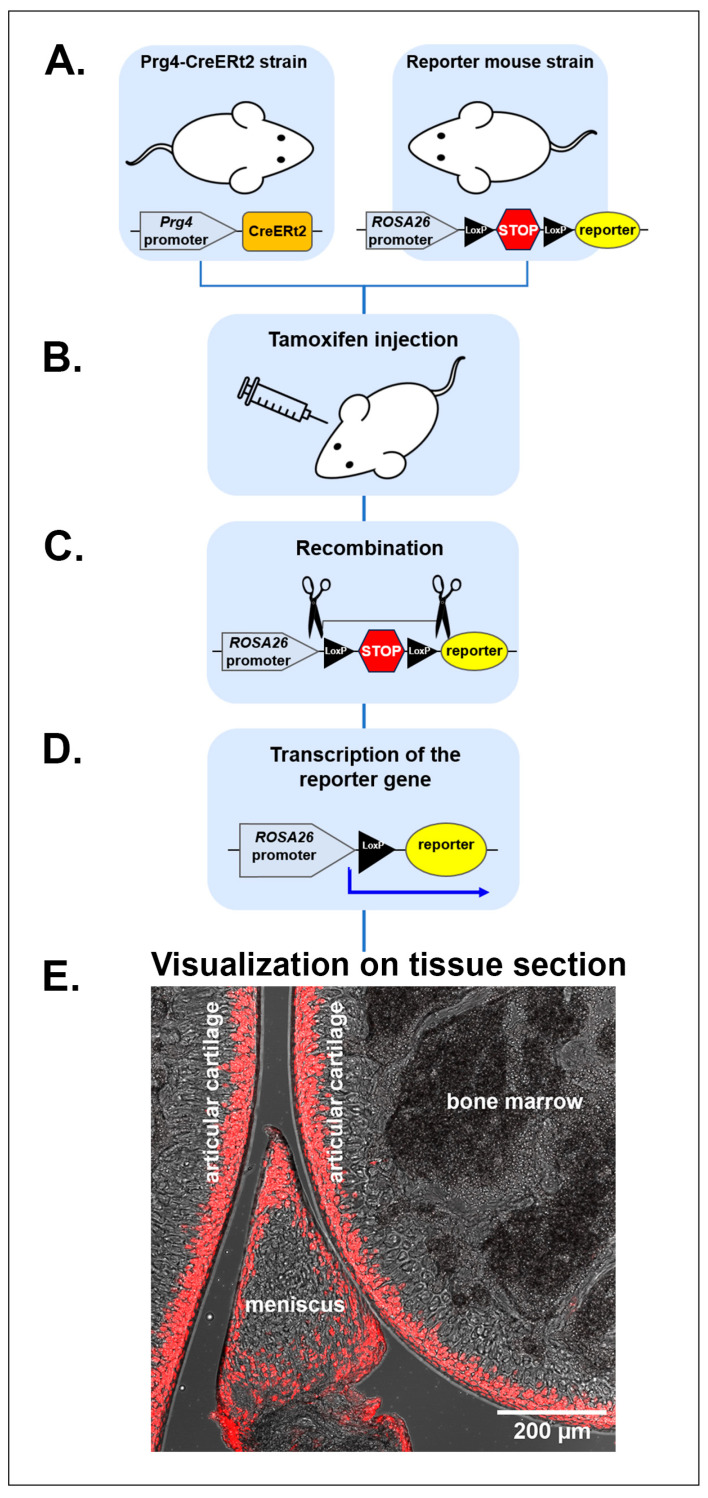
Schematic diagram indicating a ligand-dependent site-specific Cre recombinase system for fate mapping of SFZ chondrogenic progenitors in vivo. (**A**) The Prg4-CreERt2 mouse strain carries a CreERt2 cassette (orange rectangle) inserted into the translation initiation site of the endogenous *Prg4* locus. CreERt2 encodes a Cre recombinase fused to a mutant estrogen ligand-binding domain (ERt2) that requires the presence of tamoxifen, a selective estrogen receptor modulator, for activity. The Rosa26 reporter strain harbors a construct under the control of the ROSA26 promoter and a preceding LoxP-flanked transcription “STOP” sequence (red hexagon) in front of the reporter sequence (yellow oval). Blue pentagons indicate gene promoters. (**B**–**D**) Tamoxifen administration to the double transgenic Cre and Cre-activatable reporter offspring (**B**) results in Cre recombinase-mediated DNA recombination and excision of the transcriptional stop signal (**C**), followed by initiation of reporter (fluorescent protein(s)) expression (**D**). The blue arrow indicates transcription initiation. (**E**) Visualization of knee joint articular cartilage carrying genetically labeled progeny of Prg4+ SFZ cells (red), 10 days after tamoxifen injection. Representative confocal image by E.V. Medvedeva.

The development of powerful genetic tools for in vivo cell lineage tracing and fate mapping using the Cre recombinase system (detailed in Figure 1) has resulted in a greater understanding and deeper exploration of both the identity of the cells that give rise to AC and the mechanism underlying cartilage growth. The study conducted by Kozhemyakina et al. [14] traced SFZ chondrocytes by lineage labeling in AC based on their expression of proteoglycan 4 (Prg4), confirming the chondroprogenitor status of the superficial zone cells of AC for the first time. Our objective in this review was to gather all the current information about the superficial progenitors of AC.

## 2. What We Know about the Origin of Joint Cells

During the initial developmental stages of embryogenesis, the formation of joints is marked by the appearance of the interzone, a region with high cell density that consists of three layers [7,15,16]. Subsequently, at embryonic day 15.5 (E15.5) in mice, joint cavitation appears in the center of the interzone, and the tissues of synovial joints are gradually formed from the interzone cells. At the molecular level, both interzone cells and their flanking cells express a new set of genes that includes growth and differentiation factor 5 (Gdf5) [7]. Gdf5 is a member of the transforming growth factor-β (TGF-β) family and serves as a principal signaling molecule during the prechondrogenic condensation of mesenchymal cells and chondrogenesis [17]. Lineage tracing studies utilizing Gdf5-Cre mice demonstrated that cells of all mature joint components, such as articular cartilage, synovial membrane, meniscus, and intra-joint ligaments, originate from Gdf5-expressing cell lineages [18]. Using the Gdf5-CreERt2 knock-in mouse line, in 2016, the “influx model” for joint development was proposed. This model suggests that joint formation occurs through a continuous influx of new mesenchymal cells from surrounding tissues into the interzone, which contributes to the organization of joint tissue [19]. During joint cavitation at E15.5 and the subsequent formation of the articular cartilage surface, it is quite possible that the same cells shut down Gdf5 expression and turn on proteoglycan 4 (Prg4) expression (reviewed in [7,20]).

Currently, the stages of subsequent lineage specification for the different joint tissues are incompletely understood. Through lineage tracing studies, Feng et al. [21] found a population of leucine-rich repeat-containing G protein-coupled receptor 5 (Lgr5)-positive interzone cells, which were a subset of Gdf5-expressing progenitors. These cells differentiated into all internal structures of the knee joint, including the cruciate ligaments, synovial membrane, menisci, and articular chondrocytes of the joint. Within this population, cells characterized by Lgr5+/Scx-/Col22a1+ markers were identified as progenitors for the AC cell lineages and menisci [21].

It is noteworthy that the Lgr5-expressing cells discovered in the outer superficial zone of the perichondrium of the temporomandibular joint (TMJ) were found to be responsible for maintaining cartilage tissue [22]. Unlike knee articular cartilage, mandibular condylar cartilage is lined with a fibrous, perichondrium-like tissue that persists in adults. Using the TMJ as a model in multiple species, it was identified that Lgr5-expressing cells form a Wnt inhibitory niche required to maintain an appropriate pool of Wnt-inactive chondroprogenitors and thus preserve chondrocyte phenotypic identity [22]. Unlike Lgr5-positive cells in the knee joint, TMJ Lgr5-expressing cells were not considered chondroprogenitors. These cells did not retain EdU after a pulse, nor did they express aggrecan (ACAN) or type II collagen. However, induced ablation of Lgr5-expressing cells in the TMJ during the early morphogenesis stages resulted in disrupted joint formation [22].

Another progenitor cell population that gives rise to articular chondrocytes and expresses the nuclear factor of activated T-cells, cytoplasmic 1 (Nfatc1), was revealed due to the application of genetic cell-lineage tracing technology [23]. Although within the musculoskeletal system, Nfatc1 was known as a crucial regulator for osteoclast and osteoblast differentiation, the novel findings [23] reported the expression of Nfatc1 in articular, but not growth plate, cartilage throughout embryonic development and postnatal growth. Using the Nfatc1-CreERT2 mouse strain, Zhang et al. [23] demonstrated that, at the early stage of mouse knee development, Nfatc1 expressed selectively in the flanking region of the joint interzone, and a progeny of Nfatc1-containing cells could be found in almost all layers of AC in the knee joint, including the SFZ [23]. Nfatc1 expression was diminished with the differentiation of AC progenitors, and in vitro suppression of NFATc1 induced spontaneous chondrocyte differentiation [23].

## 3. Prg4-Producing Cells of a Joint

The superficial zone of AC is characterized by elongated, flat cells oriented parallel to the cartilage surface. The gene expression profiles of SFZ cells are different from those of underlying articular chondrocytes [24,25,26]. Unlike chondrocytes in transitional and deep zones, SFZ cells express stem cell markers such as CD105, CD34, and Sox2 [24] and produce Prg4/lubricin [27,28,29,30]. Comprising numerous repeated mucin-like domains [31,32,33], Prg4 plays a pivotal role in maintaining the lubricated cartilage surfaces of the joint, working in concert with hyaluronic acid and phospholipid molecules [34,35]. A shift in lubricin expression from the SFZ to the intermediate zone was noted in patients with osteoarthritis [36], and an intra-articular injection of PRG4 attenuated cartilage damage and reduced inflammation in a porcine model of posttraumatic osteoarthritis (OA) [37].

A recent study identified that the bone morphogenetic protein (BMP) antagonist Gremlin 1 (Grem1), a secreted protein, marked the SFZ progenitor cell population in adult mice’s AC and confirmed the loss of Grem1-lineage articular cells with age [38]. Employing the Grem1-CreERt2 mouse strain, tamoxifen was administered at postnatal days 4–6 (P4–6), and Grem1-lineage chondroprogenitor cells were immediately observed within the cartilaginous epiphysis and meniscus (about 39% of chondrocytes). In later stages of joint development, the Grem1-lineage progeny cells transdifferentiated to osteoblasts of subchondral bone during secondary ossification center formation, and by one month they populated the entire joint, including the third part of the AC [38]. However, tamoxifen injected at 6 weeks of age revealed Grem1-positive cells in the SFZ of articular cartilage only [38]. Immunofluorescence staining of tissue samples showed overlap between articular Grem1-lineage cells and Prg4-expressing progenitors (12% of the AC double positive), but the Grem1-lineage cells were largely distinct from collagen type II (Col2)-expressing chondrocytes [38]. Clustering of scRNAseq data for the Grem1 lineage revealed five distinct cell clusters [38]. Ablation of both Prg4- and Grem1-lineage populations resulted in the depletion of SFZ cells. However, the loss of the Grem1 lineage, but not the Prg4 lineage, led to histological features typical of osteoarthritis [38].

In addition to SFZ cells, the cells surrounding the joint cavity, including those of the synovium and meniscus [39,40,41,42], and other connective tissues, such as cruciate ligaments and tendons [40,43], also produce Prg4, potentially indicating the shared origin of these structures. On that note, Richard et al. made the surprising observation that tenomodulin (TNMD), a functional marker for ligament and tendon cells, is co-expressed with PRG4 in the SFZ of human fetal AC [44]. Notably, Prg4 is absent in the hyaline cartilage tissue of the epiphyseal growth plate [14,32,45], a site of longitudinal growth of the long bones [46], even though the growth plate boasts its own niche for progenitor cells [47]. 

## 4. The Impact of Prg4 Deficiency on Joints

In humans, two mutant alleles of the PRG4 gene are associated with camptodactyly-arthropathy-coxa vara-pericarditis (CACP) syndrome. CACP syndrome is a rare disease with autosomal recessive inheritance characterized by synovial hyperplasia. CACP patients’ synovial fluid is not lubricating enough to reduce friction. The typical clinical symptoms of this disorder include congenital or early-onset flexion deformity of the phalangeal joints, a reduced inclination angle of the femoral neck, and pericardial or pleural effusions. Individuals with CACP syndrome experience non-inflammatory arthropathy, which is defined by pain, swelling, and restricted joint mobility [48,49,50,51].

Mice lacking Prg4 demonstrate degeneration of the cartilage and altered skeletal morphology. Studies in Prg4-deficient animal models indicate that the joint displays a loss of cells in the superficial and upper transitional zones and disruption of collagen fibril orientation, along with non-inflammatory hyperplastic synovial membrane and subintimal fibrosis [32,51,52]. In Prg4-null mice, SFZ cells with activated caspase-3, a crucial mediator of apoptosis, decreased after an intra-articular injection of human recombinant PRG4 protein [53]. The TMJ of mature *Prg4^−/−^* mice has significantly increased OARSI (Osteoarthritis Research Society International) scores when compared to wild-type mice and, therefore, might be used in osteoarthritis modeling [22]. It is worth noting that elevated articular cartilage thickness was found in Prg4-null mouse lines, as well as abnormalities in the subchondral bone [33,54,55,56].

## 5. Regulation of Prg4 Expression in SFZ Cells

**Figure 2:**
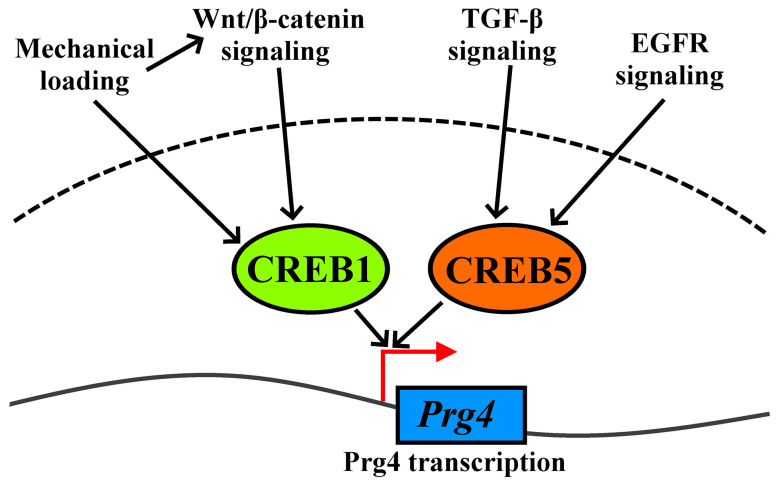
Schematic diagram indicating signaling pathways regulating Prg4 expression in the SFZ of AC. Green and orange ovals represent transcription factors; the blue rectangle represents the gene; black arrows indicate downstream activation, while the red arrow indicates transcription initiation. TGF-β, transforming growth factor beta 1; EGFR, epidermal growth factor receptor; CREB1, cyclic AMP-responsive element-binding protein 1; CREB5, cyclic AMP-responsive element-binding protein 5; Prg4, proteoglycan 4.

The growing evidence indicates that, besides joint surface lubrication, Prg4 production in SFZ cells is crucial for maintaining articular cartilage homeostasis and signaling [4]. Several pathways, including Wnt, TGF-β, and epidermal growth factor receptor (EGFR), are involved in regulating Prg4 expression in the SFZ of AC [24,57] (Figure 2). Activated TGF-β signaling and elevated Prg4 protein production in response to mechanical stimuli and shear stress loading were demonstrated both in vitro and in mature AC [58,59,60,61,62]. Elevated levels of Prg4 and TGF-β expression in the SFZ due to moderate exercise were detected, along with suppressed subchondral bone destruction in 9- and 18-month-old rat models [61].

Using a tamoxifen-inducible transgenic mouse strain, Yasuhara et al. [24] revealed that Wnt/β-catenin signaling is a key regulator of SFZ cell phenotype and proliferation. Acute activation of Wnt/β-catenin signaling led to an increase in SFZ thickness and Prg4 expression, while conditional ablation of β-catenin caused the opposite effect [24]. SFZ cells in culture maintain their phenotypic characteristics and strong expression of Prg4 under chronic stimulation of Wnt/β-catenin signaling elicited by exogenous Wnt3a [24]. Among Wnt ligands, Wnt5a, Wnt5b, and Wnt9a were highly expressed in SFZ cells of mice, and recombinant human WNT5A, WNT5B [63], and WNT16 [64] stimulated Prg4 expression in vitro. And to differentiate induced pluripotent stem cells (iPSCs) from Prg4-positive cells, the protocol included a sequential combination of Wnt3a, activin A, TGF-β1, and basic fibroblast growth factor (bFGF) [65]. Mechanical loading upregulated the expression of Wnt ligands and further promoted Prg4 transcription, probably through the initiation of the Creb1 transcription factor [63].

Recently, cyclic AMP-responsive element-binding protein 5 (Creb5) was identified as a novel transcription factor selectively expressed in Prg4-positive cells in synovial joints [25]. In AC, Creb5 was found only in SFZ cells and not in the deep zone [25]. Interestingly, forced expression of Creb5 in deep-zone chondrocytes confers competence for Prg4 expression [25]. Creb5 was required for TGF-β and EGFR signaling to induce Prg4 expression in SFZ cells. By performing the assay for transposase-accessible chromatin (ATAC-seq), it was found that Creb5 directly binds to two Prg4 promoter-proximal regulatory elements, specifically in SFZ cells [25].

It was demonstrated in vitro that lubricin activates NF-κB through toll-like receptors 2, 4, and 5 in a dose-dependent manner [66]. Exogenous Prg4 protein reduced nuclear levels of NF-κB in fibroblast-like synoviocytes (FLSs) in vitro. Alternatively, the knockout of Prg4 in FLSs led to higher nuclear levels of NF-κB in cells compared to the control [67]. Additionally, it was identified that transforming growth factor-β (TGF-β) signaling was negatively regulated by Prg4 in the SFZ of AC through the suppression of NF-κB [4,56].

## 6. The Progeny of Superficial Prg4-Positive Cell Population Forms Postnatal AC

**Figure 3:**
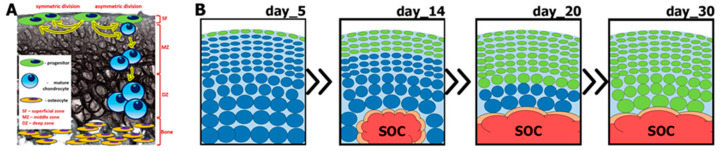
Model for postnatal development of articular cartilage. (**A**) Both symmetric and asymmetric division by progenitor cells are necessary for the population support and turnover of articular cartilage chondrocytes. (**B**) In the first month following birth, the articular cartilage tissue undergoes turnover. SOC—secondary ossification center; SF—superficial zone; MZ—middle zone; DZ—deep zone.

To evaluate the properties and specify the functions of the Prg4-expressing cell population in the SFZ, Kozhemyakina et al. [14] performed cell-tracing experiments with a novel transgenic mouse strain, Prg4-CreERt2 (Figure 1). When tamoxifen was injected at E17.5, labeled chondrocytes were observed in all layers of AC at one month of age. However, a tamoxifen injection at one month resulted in labeled cells only reaching the deep zone at 18 months of age [14]. The authors concluded that the progeny of Prg4-expressing cells of the SFZ in the developing joint at E17.5 gave rise to chondrocytes in all regions of AC. Meanwhile, the properties of the Prg4-expressing progenitor cell population changed after postnatal day 30 (Figure 3). For the first time, it was demonstrated that postnatal renewal of articular cartilage tissue occurs and that the progeny of SFZ cells are the source of chondrocytes in the underlying layers of AC [14].

To continue with the theme of this research, Decker et al. proposed to track Prg4-expressing superficial cells of AC in a slightly different way. To trace Prg4-expressing cells, the Prg4-CreERt2 line was bred with R26-Confetti reporter mice [7,68], in which individual cells were traced by random and persistent acquisition of one of four color reporters. This approach allowed for the simultaneous monitoring of the daughter cells of individual Prg4-expressing progenitors. Following a tamoxifen injection at E17.5, Decker et al. [68] described distinct, compact clusters expressing different reporters that grew with aging in postnatal animals. Of note, Prg4-Confetti-traced cell groups formed vertical columns, as observed by Kozhemyakina et al. [14]; however, those columns actually had a mixed origin [69]. Taken together, the findings from lineage tracing experiments suggest that postnatal AC development and growth involve the formation of non-daughter cell stacks. The data indicated that cell proliferation plays a role in early tissue growth. A major mechanism for the thickening of AC was provided by increases in chondrocyte volume and the local alignment of non-daughter cells to form stacks perpendicular to the AC surface [68].

The behavior of superficial AC cells was further characterized by Li et al. [69] using several lines of transgenic animals, including multiple inducible Cre strains. Firstly, they employed a mouse strain that accumulated H2B histone conjugated with green fluorescent protein (GFP) upon exposure to doxycycline. After cessation of doxycycline treatment, H2B-GFP was diluted with every division cell cycle, which allowed for the visualization of slowly dividing cells based on their GFP retention [69]. One month after the final doxycycline dose made at P2, only SFZ cells retained GFP, which confirmed that SFZ cells were slow-dividing cells and less proliferative than the chondrocytes below them [69]. Next, applying clonal genetic tracing combined with immunohistochemistry, Li and colleagues revealed that superficial cells renew their number by symmetric division, express the mesenchymal stem cell marker CD73, and generate chondrocytes via both asymmetric and symmetric differentiation [69]. Finally, the authors found that cartilage renewal occurs as the progeny of SFZ cells fully replace fetal chondrocytes during early postnatal life [69].

Mechanical stimuli are a significant factor in chondrocyte fate. Chondrocyte maturation and proliferation programs are governed by intracellular calcium signaling, which is one of the significant factors in the responses of chondrocytes to physical stimuli [70,71]. Biomechanical stress is capable of increasing the expression of the calcium-sensing receptor (CaSR), a member of the G protein-coupled receptor family, which is a pivotal driver of chondrogenic terminal differentiation in cultures of TMJ chondrocytes [72]. CaSR has been reported to be expressed in the SFZ of TMJ cartilage [73]. A recent study revealed that CaSR, in addition to promoting cell differentiation in mature chondrocytes, mediated the function of biomechanically promoted parathyroid hormone-related peptide (PTHrP) gene expression [73], hereafter taking on a role in the proliferative behaviors of SFZ cells [74]. The activation of the PTHrP nuclear localization sequence by CaSR was shown to increase the proliferation of SFZ cells [74]. Ablating PTHrP in the Prg4-expressing SFZ cells suppressed proliferation but showed no impact on CaSR expression, and the pharmacological activation of CaSR in PTHrP-ablated animals could not stimulate proliferative responses [74].

To examine the mechanism of SFZ progenitor differentiation, Maenohara and colleagues utilized homozygous *Prg4^CreERt2/CreERt2^* (tamoxifen-inducible Prg4-knockout) mouse strain [56]. Eight weeks after tamoxifen administration at P7, the authors observed abnormally thickened AC, and the cells in the SFZ disappeared [56]. Ex vivo confirmation of the results was obtained using femoral heads from 3-day-old mice. Following 3 weeks of culture, the SFZ disappeared in the femoral heads, similar to what was observed in vivo in the joints. Ectopic endochondral ossification was indicated in the knees of Prg4-null mice with age [56]. The authors observed labeled progeny of Prg4-deficient cells in the SFZ and middle zone of the AC one week after tamoxifen injection. As predicted, in a control group, Prg4-positive cells were restricted to the SFZ [56]. These data indicated that the loss of function of lubricin/Prg4 led to the abnormal expansion and differentiation of SFZ cells, and Maenohara et al. proposed that lubricin may suppress the chondrogenic differentiation of SFZ progenitors [56], maintaining the structure of articular cartilage and preserving the population of progenitor cells in the superficial zone. In summary, accumulating evidence indicates that AC-resident Prg4-positive cells may function as unipotent stem cells whose proliferation is constrained in adulthood.

## 7. Regenerative Potential of Adult Superficial Progenitors of Articular Cartilage In Vivo

Hyaline cartilage lesions are replaced by fibrocartilage, which is structurally and physiologically inferior [8,10], underscoring the pressing need for understanding the molecular mechanisms of cartilage healing. The regenerative potential of chondroprogenitor cells in adult AC tissues is a topic of debate. Prg4-producing cells inhabit both the SFZ of cartilage and the synovial membrane [20,75,76]. Using the osteochondral defect model, Decker and collaborators [68] showed that the injury site was filled by the progeny of Prg4-positive cells within 7 days after surgery. The authors concluded that the progeny of Prg4-positive cells migrated from the synovial membrane, which had enlarged into the synovial joint in response to an acute knee cartilage injury [68]. Although a massive expansion of synoviocytes was established following a knee injury [77], Chagin et al. [75] accurately pointed out that Prg4 was not a specific marker for synovial cells.

A recent report by Massengale et al. [45] advanced the understanding of the contributions of Prg4-positive cells to cartilage healing in a model of full-thickness cartilage injury, which provides access of bone marrow stromal cells to the defect [45]. The authors used inducible mouse strains containing Cre genetic constructs under the promoters of the genes encoding Prg4, aggrecan (a chondrocyte marker), and transcription factor Sp7 (a marker of adult marrow stromal cells) [45]. In line with prior studies, Massengale et al. [45] identified that neither superficial cells, which co-express aggrecan and Prg4, nor bone marrow cells, which produce Sp7, predominate in cartilage repair following injury. Instead, reparative Prg4-positive populations migrate to the wound bed from the periarticular soft tissue [45]. 

Zhang et al. [78] used a murine genetic modification (Prg4-CreERt2_R26mTmG/DTA mouse) that induces Prg4-positive cell death by diphtheria toxin A (DTA) injection and surgical destabilization of the medial meniscus (DMM) to damage the cartilage surface in order to determine whether SFZ cells have the ability to regenerate cartilage. Sham or DMM surgeries were performed on 10-week-old mice that had or had not previously undergone DTA ablation. After 12 weeks, the knee joint was examined. Surprisingly, the mice whose surface chondrocytes had undergone DTA ablation before DMM showed significantly less cartilage damage than the control mice that underwent DMM [78]. Researchers concluded that living surface chondrocytes enhanced cartilage damage after DMM, even though these cells ultimately died as a consequence of DMM [78]. However, the possibility cannot be ruled out that this result was a consequence of DTA ablation of Prg4-positive cells in the synovial membrane, which are well known for initiating joint inflammation in response to injury.

Therefore, to date, there is no in vivo evidence that cartilage SPZ progenitor cells play a role in adult cartilage regeneration. However, current studies focus on investigating the regenerative potential of SFZ cells.

Non-surgical models are superior for simulating athletic injuries characterized by acute, extensive AC damage. Similar to invasive injury models, non-surgical cyclic compression on the joint of living rats results in a localized condyle lesion [79]. Live/dead staining demonstrated chondrocyte death in the superficial cartilage within 6 h due to direct compressive loading (20N) [79]. There was a decrease in the intensity of superficial Prg4-positive immunostaining in the damaged area immediately after loading compared to the non-loaded region [79]. Decreased superficial cartilage Prg4 was in line with prior experiments both in vivo [80] and ex vivo [59]. Four weeks after compression, Prg4/lubricin distribution gradually recovered to normal levels in the loading-damaged AC [79]. In contrast to the surgical model of OA based on cartilage surface damage to the knee joint, in vivo low-dose cyclic compression, a non-surgical model, demonstrated more optimistic outcomes [79]. Probably, the extent of cartilage tissue restoration may be contingent upon the nature and severity of the damage.

The barrier to chondrocytes’ regeneration ability may be due to their inability to migrate through the dense collagen matrix of cartilage tissue. In a recent study [81], chondrocyte migratory activity was evaluated after enzymatic treatment followed by 1–2 weeks of culturing of a cartilage explant from porcine femoral condyles. Embedding the cartilage explant within the collagen gel allowed for the visualization of the chondrocytes infiltrating the gap. The migration of cells from the SFZ in the enzymatically treated porcine cartilage explant was found to be higher compared to that of chondrocytes from the deeper zones [81]. In the absence of enzyme treatment, there was no migration of chondrocytes out of the cartilage explant within the gap. Of note, SFZ cells no longer displayed Prg4-positive staining upon migration [81].

## 8. Conclusions

The cell population present in the superficial zone of articular cartilage produces lubricin/Prg4 to decrease the coefficient of friction between articular surfaces during joint movement. Besides its lubrication function, Prg4 contributes to maintaining the homeostasis of articular cartilage tissue as well as preserving the superficial progenitor population. SFZ progenitors comprise slowly dividing and renewing cells, serving as a source for the renewal of articular cartilage tissue during postnatal life.

In vivo cell lineage tracing studies have demonstrated that Prg4 is a specific progenitor marker in mice. Taking into account all the studies using the genetic tracing method reviewed, it is important to keep in mind that the genetic tracing method has some pitfalls. First of all, there is the so-called ‘leakage’, when the cell begins to produce a fluorescent protein reporter randomly due to stochastic events. This occurs more frequently with the Confetti reporter construct. Secondly, the success rate of recombination is not 100%, and the number of cells recombinated is linked to the number of tamoxifen injections administered. It is impossible to determine which portion of the cells has gone through recombination and which has not.

Playing a role in multiple signaling pathways, Prg4 regulates the differentiation of SFZ progenitors and supports the SFZ structure. The disappearance of SFZ cells due to Prg4 depletion is associated with the formation of ectopic ossifications in tissues. Prg4-positive cells located in the synovial membrane and ligaments also require additional studies. It is not clear what triggers the expression of lubricin in cells outside the articular cartilage. The cause of lubricin expression in cells outside the articular cartilage remains unclear, as these tissues are not under the same mechanical load as cartilage.

In the postnatal period, the surface cells of articular cartilage are active in producing progeny, but they stop renewing cartilage tissue after one month of age, as is known. However, this cell population can still be found in the superficial part of articular cartilage even in old age. It seems very exciting to be able to unravel the molecular mechanism for switching the behavior of the superficial cell population and find a way to return the superficial progenitors of adult articular cartilage to a state where they can actively produce progeny for tissue renewal. Joint regenerative medicine research on superficial progenitors in the coming decade will be clearly directed towards this target. Understanding the mechanisms underlying hyaline cartilage tissue formation and homeostasis may facilitate the development of strategies for effective joint regeneration, which continues to be one of the primary challenges in the field of orthopedics.
